# The Use of Procalcitonin in the Diagnosis of Acute Appendicitis: A Systematic Review

**DOI:** 10.7759/cureus.30292

**Published:** 2022-10-14

**Authors:** Lucy Dale

**Affiliations:** 1 Surgery, Ninewells Hospital and Medical School, Dundee, GBR

**Keywords:** appendix histopathology, biochemical marker, procalcitonin, complicated appendicitis, uncomplicated appendicitis

## Abstract

Background: Acute appendicitis (AA) is one of the most common surgical pathologies. Its diagnosis is often carried out based on clinical signs and symptoms, with additional minimally invasive tests (i.e., blood testing) done to support the diagnosis. Procalcitonin (PCT) is a relatively novel biomarker that is starting to be used by clinicians for patients admitted into hospitals with a variety of infections. Its level can be used to identify the presence of infection. The aim of this review is to assess how useful PCT is as a biomarker in supporting clinicians' assessment of patients with suspected appendicitis.

Methods: A systematic literature search was carried out, yielding a total of 16 primary research papers deemed appropriate for appraisal.

Results: The usefulness of PCT in aiding the diagnosis of AA depends on the severity of appendicitis. Patients who experience complicated appendicitis (CAA) such as perforation, gangrene, or necrosis have a significantly raised PCT level (p<0.05) compared to those with uncomplicated appendicitis (UAA) and a variety of other non-appendiceal intra-abdominal pathologies.

Conclusions: The use of PCT in UAA is weak, however, PCT was deemed useful in helping predict CAA, thus helping portray the severity of infection. This, in turn, will help ensure patients are taken to the operating theatre in a timely and safe manner for subsequent appendicectomy.

## Introduction and background

The appendix

The appendix is a small vestigial organ located distally to the ileocecal valve [1]. Also referred to as the "Vermiform appendix," this structure was first described in the 16th century by the Italian anatomist, Berengario de Capri [2].

The appendix is roughly 2-4 inches long and is most commonly situated in the retrocecal position, although this position varies in individuals [3]. It is embryologically derived from the midgut and medially rotates throughout gestation to end up residing in the right iliac fossa. The arterial supply of the appendix comes from a branch of the ileocolic artery, with venous drainage from the appendicular vein [4].

Whilst the appendix is a vestigial organ, its usefulness in relation to its function has been widely debated. Some scholars believe that this organ is of no immunological benefit to the human body, whereas others believe that it is. This is because the appendix houses an array of gut microbiota. Therefore, this serves as a protective mechanism, which is particularly useful when patients experience gastrointestinal infections [5]. In essence, the appendix microbiota serves as a "replenishment" for those used up in fighting active infection.

**Figure 1:**
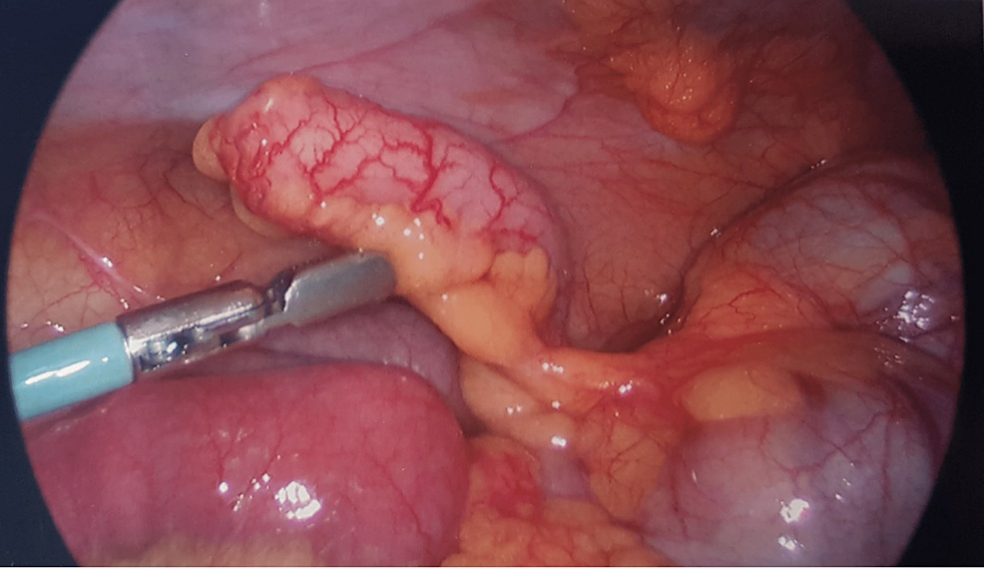
Inflamed appendix on laparoscopy Joseph CT, Tsang CLN, Goltsman D, Garibotto NL, Mekisic A. Synchronous Acute Appendicitis and Cholecystitis in a Paediatric Patient with Salmonella Enteritis. Cureus. 2020;12(3). doi:10.7759/CUREUS.7214

Regarding pathological processes, inflammation of the appendix-i.e., "appendicitis." The incidence of appendicitis is variable. It most commonly affects young people aged between 10 and 20, but the older population can be susceptible to appendicitis too [6]. The aetiology of appendicitis is multifaceted; it can be due to the infiltration of appendix tissue by bacteria or viruses (Figure 1) [7]. Alternatively, appendicitis can occur due to impacted stool at the level where the appendix joins the caecum, thus resulting in inflammation and subsequent infection [1].

The presentation of appendicitis can be variable. The "classical presentation" of appendicitis is pain that starts off in the umbilical/peri-umbilical area of the abdomen and migrates down to the right lower quadrant. In reality, a number of patients present with generalised symptoms including nausea, vomiting, and fever. Clinical signs can include a Rovsing sign (RIF tenderness experienced when palpating the LIF) and a Psoas sign (pain in the RIF when the right hip is extended backwards) [8]. Both of these clinical signs have been documented to have good specificity for appendicitis, however, lack sensitivity [9].

**Figure 2:**
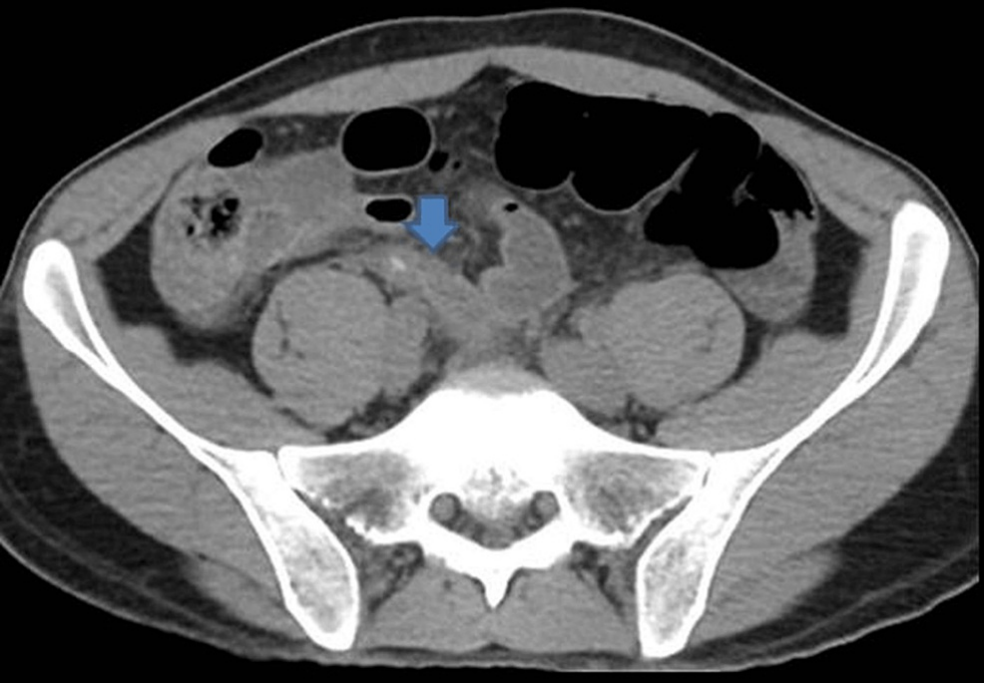
Axial, non-contrast CT scan showing acute appendicitis. Blue arrow shows a calcified deposit within the appendix. Ali M, Iqbal J, Sayani R. Accuracy of Computed Tomography in Differentiating Perforated from Nonperforated Appendicitis, Taking Histopathology as the Gold Standard. Cureus. 2018;10(12). doi:10.7759/CUREUS.3735

The majority of patients with appendicitis are diagnosed clinically, but additional tests are done by clinicians to help support the diagnosis if there is uncertainty [1]. Upon admission into the hospital, patients should receive basic investigations, including basic observations, ECG, and blood sugar measurements. A large proportion of patients will also receive blood tests to assess levels of potential inflammation/infection within the body and also to check for renal/liver function. In certain cases, if the diagnosis of appendicitis is uncertain, an ultrasound scan or CT scan of the abdomen and pelvis is requested (Figure 2) [10]. However, as mentioned at the start of this paragraph, the majority of appendicitis cases can be diagnosed just based off the clinical presentation.

The treatment for any condition should be patient-centered and patient-specific. All patients should receive appropriate analgesic and antiemetic medication should they need it. Specifically, for appendicitis, the large majority of patients will receive surgical removal of the appendix either via a laparoscopic or open approach [11]. Laparoscopic appendicectomies are often preferred because this surgical technique has been shown to reduce postoperative pain and wound infection risk. Many clinicians will still operate on patients with subacute appendicitis (often deemed to be a less severe version of acute appendicitis). However, for patients where surgery is deemed inappropriate, a course of intravenous antibiotics is often offered to help reduce infection [12].

Procalcitonin

Procalcitonin (PCT) is a type of hormone that is produced by the thyroid parafollicular cells (C cells) [13]. This hormone is then cleaved to form calcitonin, which is a hormone that primarily works to lower the body's calcium levels. The usefulness of the PCT has not truly been appreciated until recently. Increased levels of PCT have been linked to infectious processes, particularly bacterial ones [14]. PCT has also shown its usefulness in relation to the COVID-19 pandemic; since COVID-19 does not cause a high level of PCT, it is useful to clinicians in delineating whether patients potentially have a superimposed infection on top of their existing COVID infection. This can help guide appropriate antibiotic use [15].

This review is specifically going to look at the role and usefulness of PCT testing in appendicitis. It will be interesting to see whether PCT is useful in aiding appropriate further management - for example, identifying patients that need to be taken to the theatre quickly.

## Review

Materials and methods

Systematic Search

**Figure 3:**
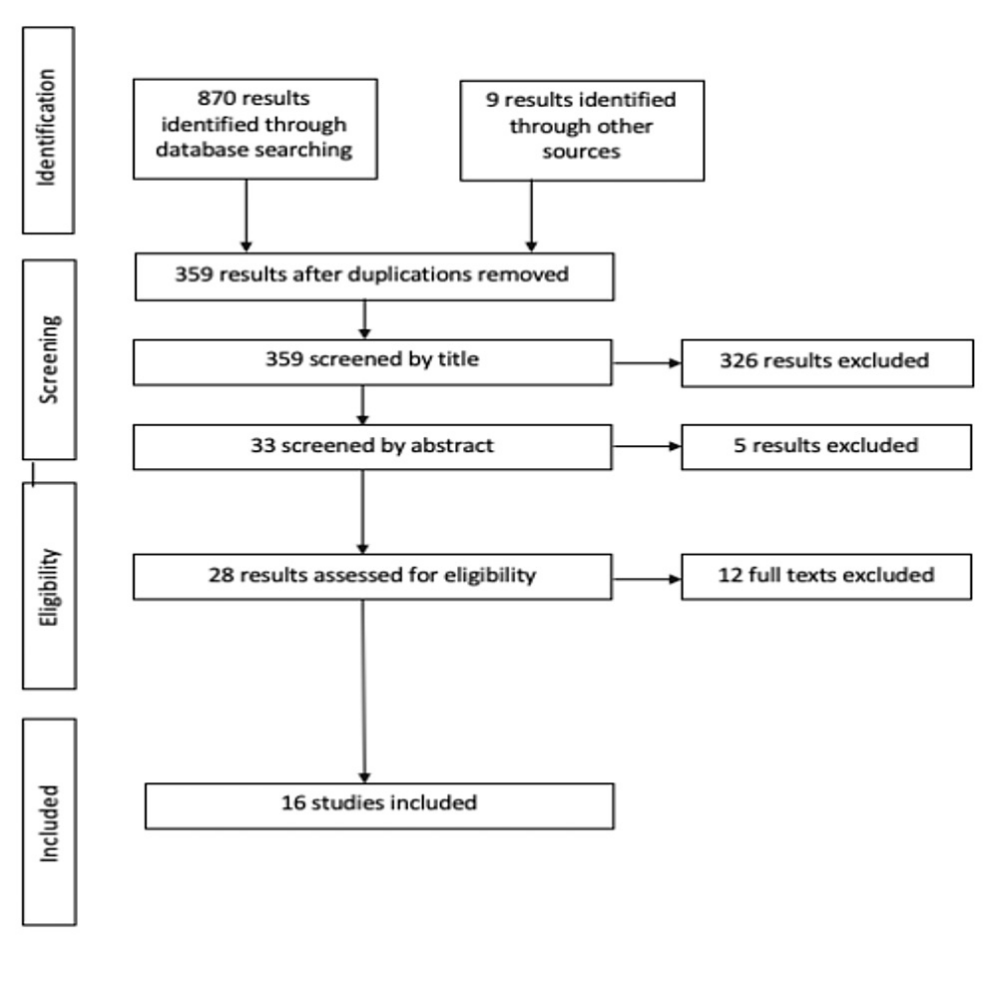
PRISMA flow chart Moher D, Liberati A, Tetzlaff J, Altman DG. Preferred reporting items for systematic reviews and meta-analyses: the PRISMA statement. *BMJ*. 2009;339(7716):332-336. doi:10.1136/BMJ.B2535

The Preferred Reporting Items for Systematic Review and Meta-Analysis (PRISMA) tool was used in this review to find appropriate papers for critical analysis (Figure 3) [16].

The databases searched included PubMed and EMBASE. Keywords included "Procalcitonin OR PCT OR Appendicitis." A search filter was applied, meaning only literature published after the year 2000 was used. All articles generated from this search were imported into Mendeley Reference Manager, and duplicates were identified and removed.

Then, all articles were screened by title, then abstract, and then full text. Specific inclusion and exclusion criteria were to be met to qualify for analysis (Table 1). All the papers chosen looked at outcomes surrounding the use of PCT in the diagnosis of AA.

**Table 1:** Eligibility criteria

Inclusion	Exclusion
Published AFTER the year 2000	Non-appendicitis intra-abdominal infections
Primary research papers	Systematic review/meta-analysis papers
0-100 years old (i.e., any age)	Non-human subjects
Human subjects	Non-English language
English language	

This yielded a total of 16 papers that were deemed appropriate for analysis (Table 2).

**Table 2:** Study characteristics CRP: C-reactive protein, PCT: procalcitonin, LF: lactoferrin, CAA: complicated acute appendicitis, PA: perforated appendicitis, ED: emergency department, NP: neopterin, AP: appendicitis, UAA: uncomplicated appendicitis, WCC: white cell count, N/L ratio: neutrophil/lymphocyte ratio, IL-6: interleukin 6.

Authors	Study type	Primary objective	Sample size	Year	Age group (of appendicitis patients)	Summary of conclusions
Abbas et al. [17]	Comparative	Investigate value of SAA and PCT compared to CRP in the diagnosis of AA	147	2014	Mean age 36 (±17) years	PCT supersedes alternative inflammatory markers in the diagnosis of AA.
Chandel et al. [18]	Observational	Comparing the diagnostic value of PCT to other parameters including CRP in AA	28	2011	Up to 15 years	PCT is useful in the diagnosis of AA in combination with clinical signs and symptoms and its use could help prevent unnecessary appendicectomies.
Dal et al. [19]	Prospective cohort	Alvarado score combined with the use of the biological indicators of CRP, PCT and NP in the diagnosis of AA	100	2019	Mean age 32.8 (±13.7) years	When considered alone, PCT is not useful in aiding AA diagnosis. However, it can be used in distinguishing UAA from CAA.
Gavela et al. [20]	Comparative	Evaluate the use of PCT and CRP on admission as predictors of the severity of appendicitis in children	111	2012	Median age 10 (range, 3.1–17.1 years) in group 1 and 7.3 (range, 1.2–13.5 years) in group 2	Patients with an increased PCT have a higher risk of having CAA therefore must be looked after closely.
Haghi et al. [21]	Prospective cohort	Find the diagnostic values of procalcitonin and IL-6 in diagnosing acute appendicitis	80	2018	Frequency of patients in the 0–20, 21–40, 41–60, and older than 60 years old age groups were 30 (37.5%), 39 (48.8%), 6 (7.5%), and 5 (6.2%) patients, respectively	PCT can be used alongside other biomarkers usefully in ruling out AA and reducing negative appendicectomy rates.
Kafetzis et al. [22]	Comparative	Assess the diagnostic value of PCT in 212 children with appendicitis and compare it with that of the standard diagnostic modalities and abdominal ultrasound findings	212	2005	Mean age 9.5 (±2.8) years – group 1/no pathological findings 10 (±2.4) years – group 2/reactive follicular hyperplasia of appendix 9(±2.2) years – group 3/acute appendicitis 8(±3.2) years – group 4/acute appendicitis and perforation 10.5(±2.2) years – group 5/acute necrotizing appendicitis	PCT can be a useful biomarker in helping diagnose CAA.
Kaya et al. [23]	Prospective cohort	Evaluate the diagnostic utility of D-dimer, PCT and CRP measurements in the acute appendicitis	78	2012	Mean age 25.4 (±11.1) years	PCT is no better at helping diagnose AA in comparison to other biomarkers
Khan et al. [24]	Prospective cohort	Assess the predictive value of procalcitonin in detecting acute AP in children	50	2012	Mean age ﻿11.25 (±3.13) years	PCT can be a strong predictor of AA.
Li et al. [25]	Case-control	Assay the variation of PCT in adult patients with uncomplicated and complicated acute appendicitis	336	2020	18-83 (38.82 ± 17.51) years	Serum PCT could help decide treatment paths for patients diagnosed with AA.
Motie et al. [26]	Cross sectional	Evaluate the diagnostic value of serum LF and PCT in detection of patients with acute appendicitis	131	2018	Mean age 26.32 (±10.67) years	PCT is not considered to be a useful screening tool in the diagnosis of AA.
Naqvi et al. [27]	Secondary analysis	Determine levels of seven inflammatory protein mediators previously associated with intra-abdominal inflammation in a cohort of children with suspected appendicitis	185	2019	5–17 years	PCT is useful in differentiating patients with UAA from CAA.
Orellana-Henriquez et al. [28]	Observational	Determine the usefulness of known biomarkers (including PCT) as pre-operative predictors of CAA and PA	128	2020	Median age 30 years	PCT is useful in ruling out PA when AA is suspected.
Sand et al. [29]	Prospective bicentre	Determine whether or not the PCT levels in the serum of patients with acute appendicitis have any diagnostic value	103	2009	Median age ± 8 SD: 33 8 ± 18.5 years	PCT may be useful in cases of CAA including perforation and gangrenous however its use in UAA is lacking.
Vaziri et al. [30]	Prospective cohort	Assess the value of procalcitonin as a predictor of diagnosis and severity of appendicitis	100	2014	Mean age 28 years	PCT is not useful in diagnosing AA but can be used as a predictor of infectious complications.
Wu et al. [31]	Prospective observational	Assess the diagnostic value of PCT in ED patients with suspected appendicitis	259	2012	Mean age 35.2 ± 18.3 years	PCT is not a useful screening tool for UAA but may be useful in screening for CAA in ED patients.
Yamashita et al. [32]	Retrospective cohort	Assess usefulness of PCT as diagnostic marker for CAA compared with BT, WCC, N/L ratio, and CRP	63	2016	Mean age 48 ± 18 years	PCT is a useful biomarker in diagnosing CAA.

Results

Paper Evidence

The literature search yielded a total of 16 papers that were deemed appropriate for appraisal. All of the studies looked at the use of PCT as a diagnostic marker in acute appendicitis. The 'New Evidence Pyramid', developed by Murad et al. in 2016, was used to identify the levels of evidence each paper presented [33]. As this review included primary research only, ‘level 1’ evidence papers (i.e., systematic reviews/meta-analyses) were not included.

The majority of papers appraised consisted of ‘level 3’ evidence, including case-control, retrospective cohort, and comparative studies. Some ‘level 2’ evidence papers were also included; these were prospective observational studies. It seems that there are a variety of different study designs used by authors when looking at the topic of PCT and its usefulness in appendicitis. Of course, ‘level 2’ evidence is preferred as it can be tailored specifically to outcomes; nevertheless, the use of ‘level 3’ evidence must not be discredited as this subject remains novel.

Patient Cohort 

In order to generate reproducible results that can be made generalisable to the population, authors must set out inclusion and exclusion criteria within the methodology section of their study [12]. When looking at PCT and its use in appendicitis, the questions one must ask regarding inclusion/exclusion criteria is whether the authors have introduced alternative patient variables into the data, thus impacting results.

Some studies did not mention inclusion/exclusion criteria for patients [18,20,22,24], with a few choosing to exclude one or two variables in their exclusion criteria (for example, pre-diagnosis antibiotics). On the other hand, some authors were found to be more stringent in their selection of patients. Orellana-Henriquez et al. [28] specifically stated that patients who have co-morbidities, co-infections, or physiological states (e.g., pregnancy) that raise inflammatory markers were to be excluded. This level of detail enables the reader to understand and appreciate how the authors have tried to reduce the number of confounding factors that have an impact on PCT results. This, in turn, helps to increase the external validity of the study.

Sample Sizes

Sample sizes for the studies were variable (range 28-336; mean 131.9). Chandel et al. [18] used 28 participants, whereas Li et al. [25] used 336 participants. Six of the studies had ≤100 participants [18,21,23,24,30,32]. The significance of this is that a smaller sample size lacks statistical power, resulting in weak internal/external validity. This makes it difficult to generalise results [34]. It is important to consider the logistical and practical variables of the studies conducted (e.g., availability and cost of PCT testing, appendicitis incidence, timeframe). Therefore, sample sizes ≥100 participants could be deemed adequate. Nevertheless, the wish for a larger sample size will always remain present as this boosts the results' validity and applicability.

Methodology

The papers analysed were a mix of qualitative and quantitative research. Of the qualitative research, observational or comparative studies were seen. The disadvantage of this study design is that there is an increased risk that confounding variables are present, thus influencing the reliability of the results. This makes it challenging to delineate the relationship between PCT and acute appendicitis. Quantitative papers included prospective cohort studies, enabling patients to be followed up over a period of time. The benefit of this is that causal links can be identified between exposure and outcome. Specifically, patients whose PCT levels were taken before the diagnosis of acute appendicitis was made via histology.

Bias

Selection bias seemed to be the main weakness of the papers selected for this study. Selection bias occurs when study participants have significantly different characteristics than those who were not chosen [35]. Selection bias is, however, removed in prospective cohort studies as researchers do not know outcomes - i.e., the levels of PCT or confirmed diagnosis of appendicitis through histology. Unfortunately, the observational studies looked at within this paper specifically chose participants to be included in their study - making the element of selection bias strong. The issue with selection bias is that the results are not representative towards the general population.

PCT and AA: The Relationship

This review proposes that PCT may prove useful in the diagnosis of complicated acute appendicitis (CAA). However, its use in uncomplicated acute appendicitis (UAA) appears weak.

All of the papers diagnosed appendicitis in patients who received an appendicectomy via histopathological examination [17-32]. This is the gold standard method for accurately diagnosing appendicitis; therefore, all authors must be praised for this. Nevertheless, Wu et al. [31] also chose to include patients who did not undergo surgical intervention and used CT imaging to aid in diagnosis. The issue with this is that CT scanning for acute appendicitis has been shown to have a sensitivity of roughly 80-90% [36]. Therefore, there is the concern of potential misdiagnoses which could skew results.

When assessing the diagnosis of appendicitis further, some papers choose to grade the tissue in relation to severity. For example, Yamashita et al. [32] divided appendiceal tissue into groups based on mural architecture, which directly correlates to the level of inflammation. This is useful because there are different levels of acute appendicitis patients presenting with UAA or CAA [37].

When comparing PCT levels in those diagnosed with acute appendicitis compared to those with nonappendiceal abdominal pathologies (e.g., urinary stones, gastroenteritis), the conclusion that can be drawn is that the levels of PCT were, on average, higher in the acute appendicitis patients [17,24]. However, the statistical significance of this was variable. For example, Naqvi et al. [27] quoted a P<0.0001 difference in PCT for patients with appendicitis vs. non-appendiceal pathologies, whereas other authors disagreed. Most concluded that PCT was no better at predicting the diagnosis of UAA compared to other intrabdominal pathologies. This, therefore, provides thus, no influence in the long run, specifically towards which treatment the patient receives.

Some authors also looked at patients with differing clinical severity of AA. As mentioned before, appendicitis can range from uncomplicated to complicated tissue pathology. The papers that chose to look at PCT levels in both CAA and UAA patients showed interesting results.

All authors came to the conclusion that the level of PCT was higher in patients with CAA compared to UAA. From a statistical perspective, it can be concluded the level of PCT was significantly different (p<0.05) when comparing UAA to CAA.

Moreover, some authors concluded that the difference in PCT levels for CAA vs. UAA holds real-life clinical significance. That is, PCT may be useful as a predictive biomarker and highlight to clinicians the potential severity of appendicitis a patient is presenting with. This, in turn, influences the speed of treatment (e.g., appendicectomy) patients receive and their overall prognostic outcome.

## Conclusions

Overall, the literature seems to suggest that whilst PCT is not useful in the overall diagnosis of AA, it can be used by clinicians to help predict patients that have potential CAA (such as perforation, gangrene, or necrosis) compared to those with a UAA. The importance of this is that, alongside other biomarkers, PCT may help clinicians decide how quickly to take patients to the operating theatre. This will ultimately save time and prevent complications from worsening and causing systemic inflammation influencing morbidity and mortality.

However, it must be said that the current literature available on this topic is somewhat scarce and is lacking when it comes to sample size and overall hierarchy of evidence. Regarding future research, it would be useful to have a succinct randomised control trial to look at the relationship between appendicitis and PCT.
